# SIRT1 Modulators in Experimentally Induced Liver Injury

**DOI:** 10.1155/2019/8765954

**Published:** 2019-06-02

**Authors:** Hassan Farghali, Mighty Kgalalelo Kemelo, Nikolina Kutinová Canová

**Affiliations:** Institute of Pharmacology, 1st Faculty of Medicine, Charles University in Prague, Albertov 4, 128 00 Prague 2, Czech Republic

## Abstract

This article is directed at highlighting the involvement of the endogenous stress sensor SIRT1 (silent information regulator T1) as a possible factor involved in hepatoprotection. The selective SIRT1 modulators whether activators (STACs) or inhibitors are being tried experimentally and clinically. We discuss the modulation of SIRT1 on cytoprotection or even cytotoxicity in the liver chemically injured by hepatotoxic agents in rats, to shed light on the crosstalk between SIRT1 and its modulators. A combination of D-galactosamine and lipopolysaccharide (D-GalN/LPS) downregulated SIRT1 expression, while SIRT1 activators, SRT1720, resveratrol, and quercetin, upregulated SIRT1 and alleviated D-GalN/LPS-induced acute hepatotoxicity. Liver injury markers exhibited an inverse relationship with SIRT1 expression. However, under subchronic hepatotoxicity, quercetin decreased the significant increase in SIRT1 expression to lower levels which are still higher than normal ones and mitigated the liver-damaging effects of carbon tetrachloride. Each of these STACs was hepatoprotective and returned the conventional antioxidant enzymes to the baseline. Polyphenols tend to fine-tune SIRT1 expression towards normal in the liver of intoxicated rats in both acute and subchronic studies. Together, all these events give an impression that the cytoprotective effects of SIRT1 are exhibited within a definite range of expression. The catalytic activity of SIRT1 is important in the hepatoprotective effects of polyphenols where SIRT1 inhibitors block and the allosteric SIRT1 activators mimic the hepatoprotective effects of polyphenols. Our findings indicate that the pharmacologic modulation of SIRT1 could represent both an important move in alleviating hepatic insults and a future major step in the treatment of xenobiotic-induced hepatotoxicity.

## 1. Introduction

There are various liver diseases that spread all over the world. Several factors are contributing to these diseases. Among the most known factors are excessive alcohol consumption, liver viral infection, HIV, obesity that leads to nonalcoholic fatty liver disease, consumption of many drugs, parasite and fungal infections, cholestatic disorders, inherited metabolic disorders, and several other reasons. Liver disease is a substantial health problem all over the world [1, 2]. For instance, hepatic diseases are the fifth well-established cause of death in the United Kingdom [3]. A major liver disease is fibrosis with high incidence in developing countries [4]. Factors as obesity epidemics contribute to the spread of nonalcoholic fatty liver disease (NAFLD), nonalcoholic steatohepatitis (NASH), fibrosis, cirrhosis, and hepatocellular carcinoma resulting in increasing the world concern at any age and ethnicity [5–7].

Historically, phytotherapy using mainly isolated purified or semipurified active constituents was applied for treating various diseases including the liver ones. Among the several examples of natural compounds are silymarin and resveratrol. The two compounds exhibited a significant hepatoprotective potential. This effect was based on their antioxidant, anti-inflammatory, and regenerative effects [8–13]. Other compounds as quercetin and curcumin possess antioxidant and cytoprotection characteristics, but their use as hepatoprotective drugs was limited [14–16]. Nevertheless, quercetin and curcumin demonstrated according to our findings hepatoameliorative effects against liver insult in experimental models [17, 18].

Therefore, during more than 2 decades ago, we were involved in finding out some of the hepatoprotective drugs that may have a common mode of action. The hepatoameliorative profiles of the extensive investigated active constituents of the flavonoid type were reviewed [19]. We suggested that there are possible common hepatoprotective mechanisms of various compounds of natural origin. One of the mechanisms seems to reduce the effects of cell oxidative stress. Indeed, oxidative stress is the main mechanism that can be induced by toxins and various environmental factors that lead to the accumulation of toxic intermediates. Moreover, cell injury due to oxidative stress is of prime importance due to its association with senescence and various diseases such as atherosclerosis, Alzheimer's dementia, and diabetes among several others. During our work, we were interested in the involvement of the endogenous stress sensor silent information regulator T1 (SIRT1) as a possible factor involved in hepatoprotection. We have used several agents to modulate SIRT1 functions and to demonstrate its potential role as a factor that plays an important role in ameliorating liver injury.

## 2. What Is SIRT1?

It is the NAD^+^-dependent protein lysine deacetylase of the sirtuin family with many physiological functions such as regulation of energy, inflammation, neuronal signaling, cell survival, DNA repair, tissue regeneration, and stress responses. As reported, the human sirtuin isoforms, SIRT1–7, are considered the attractive therapeutic site of action for several diseases like type 2 diabetes, NAFLD, neurodegenerative, and inflammatory diseases [20–22]. Potent and selective pharmacological activators and inhibitors of sirtuins, especially of the most studied isoform SIRT1, are available, and some clinical trials have been performed. The advance in comprehension of the molecular mechanisms of sirtuin modulation by these substances provides a basis for further drug development [23, 24].

Indeed, the role of sirtuins in antioxidant and redox signaling has been considerably reviewed. As reported, the significance of antioxidant and redox signaling events is regulated by critical molecules that modulate antioxidants, reactive oxygen species (ROS), or reactive nitrogen species (RNS). The imbalances in these molecules can disturb cellular functions to become pathogenic [25]. A description of the inducibility of SIRT1 and its role as the longevity factor in cytoprotection and cancer was also documented [26]. SIRT1, which is mainly nuclear protein, deacetylates histones [27] and more than fifty nonhistone targets, inclusive of DNA repair proteins and transcription factors (e.g., p53, NF-*κ*B, p65, and PGC-1*α*) [28]. In so far as the liver is concerned, SIRT1 activation alleviates cholestatic liver damage in a cholic acid-fed mouse model of cholestasis. Therefore, it was suggested that the use of small molecule activators of SIRT1 constitutes a potential new therapeutic target for cholestatic hepatic injury [29]. SIRT1 also plays beneficial roles in regulating hepatic lipid metabolism, controlling hepatic oxidative stress and mediating hepatic inflammation through deacetylating some transcriptional regulators against the progression of fatty liver diseases [21, 30]. In the field of liver transplantation surgery, Nakamura et al. [31] identified a new class of macrophages that are activated by the heme oxygenase-1- (HO-1-) SIRT1-p53 pathway. As described, the last property is involved in mechanisms of hepatic sterile inflammation and has the potential application of being a target for new therapeutic strategies in the liver transplant recipient.

It is well established that stressful injuries to cells upregulate cytoprotective pathways. Among them, SIRT1 plays a critical role. As a cellular stress sensor regulated by metabolic, genotoxic, oxidative, and proteotoxic triggers, SIRT1 impacts cell survival by deacetylating substrate proteins leading the cell towards a cytoprotective pathway. On the other hand, extreme stress situations can direct SIRT1 to lead the cell down an apoptotic pathway. In cancer cells, SIRT1 is poorly adjusted and has been featured to have a dual role as an oncogene and tumor suppressor. Recently, the ability of SIRT1 to regulate heat shock factor 1- (HSF1-) dependent induction of the heat shock response has highlighted another pathway through which SIRT1 can modulate cytoprotection [26]. At the present time, it is clear that sirtuins are emerging to be important in normal mammalian physiology and in a variety of oxidative stress-mediated pathological situations. Next investigations are required to shed more light on further mechanisms of sirtuins in maintaining redox homeostasis. Moreover, research efforts are also needed to evaluate the druggability of sirtuins in the management of redox-regulated diseases [32].

With respect to SIRT1 modulators, we focus on this review on some polyphenols that activate SIRT1 in spite of the well-known low bioavailability in experimental setups. However, some progress has been made during the last years in the area of polyphenol bioavailability. Thus, it is necessary that researchers in this field consider and integrate this information in the design of their experiments and in result interpretation. Several experiments have been carried out to study the effects in cultured cells derived from inner tissues (*in vitro*) with some polyphenols such as proanthocyanidins, which are not absorbed from intestinal barriers. At present, it is still not clear which particular polyphenols are the most protective against the various ailments. Even with the well-established properties of polyphenols in general as beneficial agents, the net clinical results are influenced by the highly variable bioavailability. Also, their biotransformation will modify the expected biological responses at the cellular levels. Another important factor which was not well studied is the evaluation of effectiveness of the conjugated derivatives and microbial metabolites of polyphenols which necessitate more research effort. In this regard, we may recognize what exactly are the active moieties. This will lead, perhaps, to the development of better polyphenolic drugs with good pharmacokinetic properties and consequently better pharmacodynamic efficiency [33]. In this review, we hypothesized that SIRT1 could potentially alleviate chemically induced liver damage. We discuss the modulation of SIRT1 on cytoprotection or even potential cytotoxicity in the liver that is chemically injured by hepatotoxic agents in rats. We shed light on the crosstalk between SIRT1 and its modulators, i.e., activators and inhibitors, to find out possible potential mechanism(s) of hepatoprotection.

## 3. SIRT1 as a Regulator of Antioxidant and Redox Signaling in Cells

Besides conventional endogenous antioxidants such as superoxide dismutase, catalase, glutathione, and glutathione peroxidase, SIRT1 has been shown to play an eminent cytoprotective role in oxidative stress via several mechanisms. For instance, it can deacetylate the forkhead box transcription factors (FOXO1, FOXO3a, and FOXO4) [34] as well as peroxisome proliferator-activated receptor gamma coactivator 1-alpha (PGC-1*α*) [22, 35] and induce the expression of numerous antioxidant enzymes [36]. FOXOs can further activate the growth arrest and DNA damage-inducible protein, GADD45, to promote genomic stability and DNA repair [37]. Furthermore, SIRT1 also deacetylates the RelA/p65 subunit of the nuclear factor-kappa B (NF-*κ*B) complex [38] and blocks consequent NAD(P)H-mediated ROS production [39]. SIRT1 can also directly inhibit p53's oxidative stress-induced apoptotic activity [40] and the proapoptotic effects of FOXO3a [41]. Together, all these events promote oxidative stress tolerance and cell survival. These attractive features have prompted an intensive search for SIRT1 activators.

## 4. Compounds Modulating SIRT1 Activity

Roughly, SIRT1-activating compounds (STACs) can be classified into natural and synthetic activators. Otherwise, they are historically divided into generations. STACs include (a) first-generation molecules such as resveratrol and similar polyphenols, (b) second-generation molecules such as the imidazothiazoles, and (c) third-generation STACs such as benzimidazoles and urea-based scaffolds [42].

### 4.1. STACs of Natural Origin

Polyphenols are large groups of phytochemicals characterized by the presence of many phenolic groups. They occur primarily in the conjugated form, with the sugar residues (monosaccharide, disaccharide, or oligosaccharide), linked to hydroxyl groups. Polyphenols are mostly found in fruits, vegetables, cereals, and beverages. They constitute one of the most numerous substances in the plant kingdom, with over 8000 phenolic compounds currently known [43]. They can be classified according to their chemical structures into 4 main groups: phenolic acids (hydroxybenzoic and hydroxycinnamic acids), flavonoids, stilbenes, and lignans.

Flavonoids are the most abundant polyphenols in the human diet, accounting for as much as two-thirds of the total polyphenolic intake. Based on the variability of the heterocycle, flavonoids can further be divided into 6 groups: flavanols, flavones, isoflavones, flavanones, and flavanols [44]. Quercetin is the most abundant dietary flavonol, with an estimated daily intake of up to 30 mg [45]. Tea is the major source of quercetin in the Netherlands and Japan, wine in Italy, and onion and apples in the United States, Finland, and Greece [46]. Although onion is not usually consumed in high quantities, it has one of the highest quercetin contents in food [47]. Stilbenes are not as widespread as phenolic acids or flavonoids in plants. Resveratrol, the best-studied stilbene thus far, is found largely in grapes [48]. Red wine, obtained from grapes, also contains a fair amount of resveratrol.

Over the last few decades, interest in polyphenols has dramatically increased for several reasons. Firstly, they have antibiotic or biostatic effects on a variety of organisms that consume plants [49]. Secondly, dating back to prehistory, polyphenol-rich plants such as *Silybum marianum* [50], *Lagerstroemia speciosa* [51], and *Prosthechea michuacana* [52] have been widely used in ethnomedicine for treatment of many ailments. Nowadays, *silibinin*, a water-soluble *silymarin* derivate, is used experimentally and clinically as a detoxifying and hepatoprotective substance [53, 54]. Thirdly, polyphenols have antioxidant properties. The ancient custom of preserving lard or chicken fat by mixing it with onion may be based on the prevention of lipid peroxidation and rancidity by quercetin [55]. Recently, numerous epidemiologic studies strongly suggest an inverse relationship between polyphenol-rich diet and many diseases. For example, resveratrol has been associated with the “French paradox,” where the low incidence of coronary heart diseases is linked to moderate consumption of red wine [56, 57]. It is believed that resveratrol minimizes the absorption of malondialdehyde, which is involved in increasing levels of low-density lipoprotein in the onset of atherosclerosis [58]. In addition, polyphenols tend to have cytoprotective roles in many other diseases associated with oxidative stress [59] such as lung cancer [60], neurodegenerative diseases [61], and age-related cataract [62]. However, the molecular mechanisms underlying the antioxidant effects of polyphenols are poorly understood. What is already known is that polyphenols can directly scavenge ROS [63]. Their hydroxyl groups are hydrogen atom donors and can reduce the synthesis of free radicals at different stages [64]. Another explanation, which is not yet fully substantiated, is the interaction of polyphenols with sirtuins.

In 2003, the ground-breaking research of Howitz et al. revealed that SIRT1 activity could be enhanced by polyphenols [65]. Several categories of plant polyphenols, like butein, piceatannol, and isoliquiritigenin, were demonstrated to activate recombinant SIRT1 and to extend the lifespan of *Saccharomyces cerevisiae*. The most effective of these STACs, activating SIRT1 more than thirteen times, was resveratrol, while quercetin stimulated SIRT1 activity by fivefold. Resveratrol prolonged cell survival under a variety of DNA damaging conditions and extended lifespan by up to 70% in *S. cerevisiae*. Resveratrol lowered SIRT1's K_m_ but exhibited no significant effect on V_max_, suggesting positive allosteric modulation. However, the notion that resveratrol is a bona fide SIRT1 activator was quickly disputed by some authors for several reasons. Firstly, an *in vitro* deacetylation assay containing FLUOR DE LYS, which is a nonphysiological fluorescent moiety, was used in Howitz's study [66]. In the absence of this fluorophore, resveratrol had no effect on acetylated peptides and could not activate SIRT1 [67]. Secondly, resveratrol is nonspecific and has few other known direct targets; the most commonly investigated being AMP-activated protein kinase (AMPK) [68]. Many of the metabolic effects of resveratrol such as enhanced mitochondrial biogenesis and fatty acid oxidation could be directly attributed to AMPK [69]. SIRT1 and AMPK mutually coexist, share many common downstream targets, and have many overlapping cytoprotective effects [70]. Whether resveratrol can directly activate SIRT1 [65], indirectly activate SIRT1 through AMPK [71], or act independently of SIRT1 [72] is still open for debate. These controversies highlight the need for more effective and selective SIRT1 activators or inhibitors to verify the potential effects of SIRT1. Thirdly, resveratrol and most other polyphenols have relatively poor oral bioavailability. Their efficacy *in vivo* could be grossly insufficient to simulate some of the effects observed *in vitro* [56, 73]. In this regard, the drug delivery system (DDS) is intended to increase the efficacy of drugs through targeted distribution and to reduce unwanted effects. Therefore, the basic principles of nanotechnology that were developed for DDS were described. Attention is paid on resveratrol as a model polyphenol with the attractive pharmacologic profile which was established in great numbers of studies and for its wide use as a supplemental therapy. Due to the complicated pharmacokinetic profile of resveratrol with its very low bioavailability in spite of high oral absorption, the effects of resveratrol are being investigated in original nanotechnology preparations of pharmaceutical formulation. We have reported data on the current *in vitro* and *in vivo* studies with resveratrol in new types of drug formulations using different nanoparticles as liposomes, solid lipid particles, cyclodextrins, and micelles [74].

### 4.2. Synthetic STACs

To address the ambiguity surrounding “resveratrol and similar polyphenols” and sirtuins, *Sirtris Pharmaceuticals Inc.* developed a number of synthetic SIRT1 activators: SRT1460, SRT1720, SRT2183, and SRT2104. They are derivatives of an imidazothiazole scaffold and are structurally distinct from resveratrol. They are up to 1000-fold more potent for SIRT1 than resveratrol [75]. They activate SIRT1 via the same K_m_-lowering mechanism as resveratrol, but with lower EC_50_ [42]. Recently, another class of chemically distinct STACs (STAC-5, STAC-9, and STAC-10), based on benzimidazole and urea scaffolds, has been discovered [76]. These third-generation drugs exhibit the same kinetics as earlier generations. Together, all these developments show that SIRT1 can be allosterically activated by a diverse group of compounds. Having been verified as specific SIRT1 activators, the field can now refocus on the key question: is SIRT1 a safe and valid druggable target? Experiments are currently underway to investigate the health benefits of these novel SIRT1 activators in a wide range of diseases.

### 4.3. SIRT1 Inhibitors

Compared to sirtuin activators, more studies have been carried out towards sirtuin inhibitors, especially in the anticancer area. Sirtuin inhibitors with various structures have been reported for SIRT1, SIRT2, SIRT3, and SIRT5 (splitomicin, sirtinol, AGK2, cambinol, suramin, tenovin, salermide, among others). Two classes of sirtuin inhibitors, nicotinamide and thioacetyl-lysine-containing compounds, can be regarded as mechanism-based inhibitors. Other sirtuin inhibitors noncovalently bind to the sirtuin active site and thus inhibit substrate linkage (e.g., *β*-naphthol-containing inhibitors and indole derivates). A number of indole constituents were discovered from high-throughput screening of 280000 molecules as selective SIRT1 inhibitors. One of these potent inhibitors, EX-527, has an *in vitro* IC50 value in the range of 60 nM to 100 nM and is cell permeable. SIRT1 blockage has been proposed in the therapy of cancer, immunodeficiency virus infections, and Fragile X mental retardation syndrome and for preventing or treating parasitic disorders [77, 78]. The only specific SIRT1 inhibitor undergoing the clinical trials is selisistat, also known as Ex-527 or SEN0014196 [23].

## 5. Experimental Models of Hepatotoxicity

Xenobiotic-induced hepatotoxicity is a particularly important research field for liver pharmacotherapy. Therapeutics continue to be pulled off the market because of the late discovery of hepatotoxicity [79]. Clinically, drug-induced hepatotoxicity is the commonest cause of acute liver failure (ALF) [80]. Although paracetamol, by far, accounts for most of the cases of ALF [81], up to 15% of the cases remain indeterminate [82]. Besides drugs, the liver is the most susceptible target organ by numerous nonmedicinal toxins, ranging from mushrooms [83] to haloalkanes [84]. Despite all these challenges and great advances in modern medicine, there are barely any hepatoprotective drugs. Perhaps, this explains why patients resort to self-medication with herbal products and “complementary and alternative medicine” is once again on the rise [85]. Among phytochemicals, polyphenols have received much attention due to their purported antioxidant, anti-inflammatory, antimicrobial, and antitumorigenic effects and their substantiated health benefits in a wide range of diseases [19]. In our institute, we have previously shown that polyphenols such as silymarin [8], resveratrol [9, 86], curcumin [17], and quercetin [18] have hepatoameliorative potential against various experimental models of hepatotoxicity [87]. However, the mechanisms underlying their cytoprotective effects in the liver are not clear, as multiple molecular targets seem to be involved. The consensus, although controversial, is that the many health benefits of polyphenols are SIRT1 dependent [88]. Hence, we used *in vivo* experimental models of chemically induced hepatotoxicity and assessed the therapeutic potential of natural polyphenols (resveratrol, quercetin) in both single-dose and repeated-dose studies. Furthermore, polyphenols were compared and contrasted to their synthetic counterparts (SRT1720). How SIRT1 responds to these drugs was investigated. We closely looked for any patterns that might exist between SIRT1 and other conventional antioxidants (catalase, bilirubin, and HO-1).

## 6. Experimental Findings

This section summarizes our findings with a discussion. In single-dose experiments, the tested drugs were administered only once, before sampling, within either 6 or 24 hours. With the repeated dose study, the drugs were administered several times, over a period of 14 days.

### 6.1. Single-Dose Studies with D-Galactosamine/Lipopolysaccharide (D-GalN/LPS): Oxidative Stress and Hepatotoxicity

**Figure 1:**
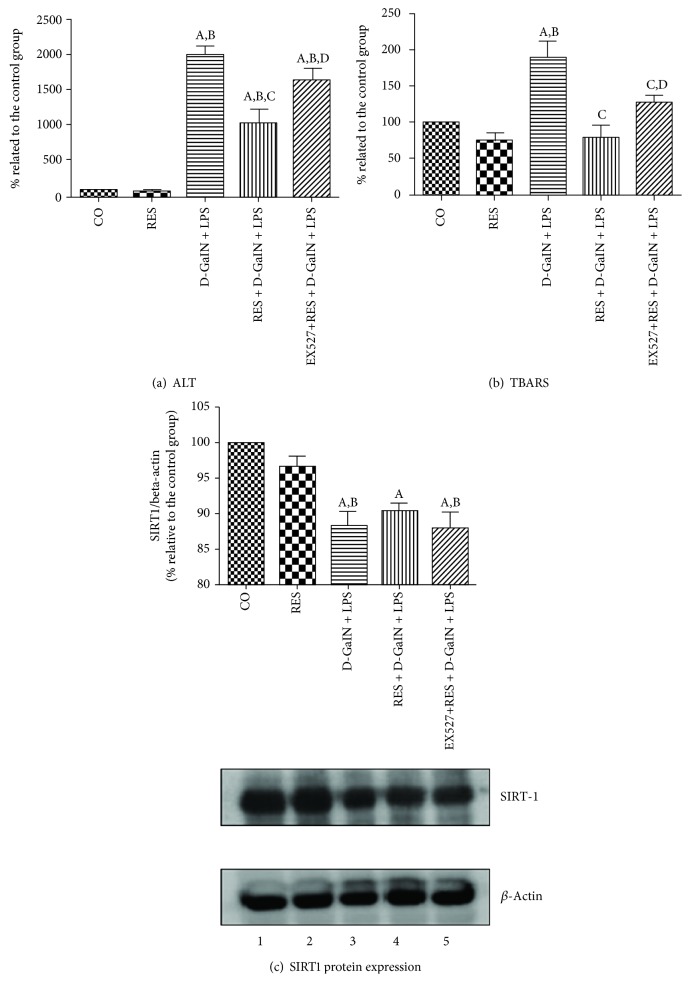
Effects of resveratrol and EX-527 pretreatment in lipopolysaccharide-induced acute hepatitis in D-galactosamine-sensitized rats (D-GalN/LPS) on the levels of (a) plasma ALT, (b) TBARS in homogenate and (c) SIRT1 expression. CO: control group; RES: 2.3 mg/kg resveratrol; D-GalN + LPS: 400 mg/kg D-galactosamine with 10 *μ*g/kg lipopolysaccharide; RES + D-GalN + LPS: 2.3 mg/kg resveratrol + D-GalN + LPS; EX − 527 + RES + D-GalN + LPS: 1 mg/kg EX-527 plus a combination of previous substances. Data are expressed as means ± SEM (*n* = 6). ^a^*P* < 0.05 versus CO. ^b^*P* < 0.05 versus RES. ^c^*P* < 0.05 versus D-GalN + LPS. ^d^*P* < 0.05 versus RES + D-GalN + LPS (courtesy of *Physiological Research*, reference [140]).

D-GalN/LPS is a well-known experimental model of hepatotoxicity. Lipopolysaccharide, an endotoxin, accumulates primarily in tissues rich in cells of the reticuloendothelial system such as the liver [89]. There, it interacts with hepatic macrophages and triggers local damage through a variety of cytotoxic mediators such as interleukin-1, tumor necrosis factor alpha (TNF-*α*), and ROS [90]. D-GalN, on the other hand, depletes the uridine nucleotide pool, inhibits protein synthesis in hepatocytes, and sensitizes the liver to the cytotoxic effects of LPS [91]. A cocktail of these drugs produces extensive liver damage that is consistent with acute liver failure seen clinically [92]. In our experimental studies, 400 mg/kg of D-GalN and 10 *μ*g/kg of LPS markedly elevated transaminases (ALT and AST) in plasma (Figure 1(a), Table 1). Interestingly, D-GalN/LPS had the greatest effect on ALT than AST [93], hence the plummet in the De Ritis (AST : ALT) ratio [94]. Although this ratio is not precise, it is used as a clinical aid in differential diagnosis of some liver pathologies. For instance, in humans, it is usually <1.0 in acute viral hepatitis, >2.0 in alcoholic hepatitis, and >1.0 in fibrosis/cirrhosis [95]. D-GalN/LPS also consistently increased conjugated dienes and/or thiobarbituric acid reactive substance (TBARS) levels in liver homogenate (Figure 1(b), Table 1), indicating excess production of ROS that can destroy hepatic macromolecules.

**Figure 2:**
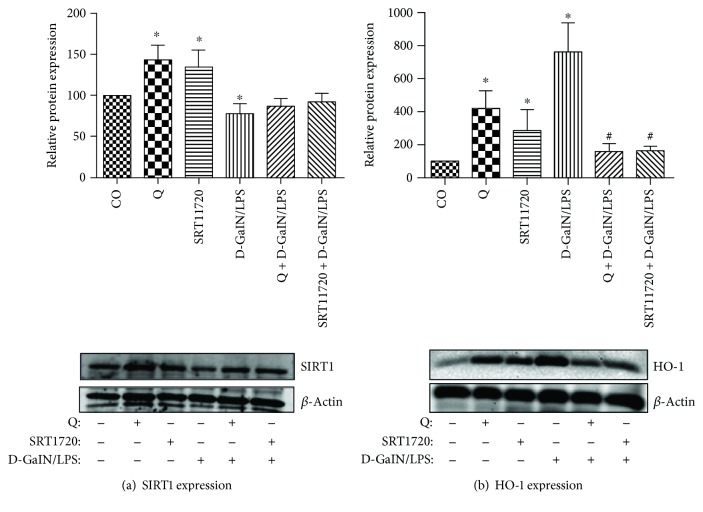
Effects of quercetin and SRT1720 pretreatments on (a) SIRT1 and (b) HO-1 protein expressions in lipopolysaccharide-induced hepatitis in D-galactosamine-sensitized (D-GalN/LPS) rats after 24 hours. Beta-actin was used as an endogenous control. CO: negative control, vehicle only; Q: quercetin 50 mg/kg; SRT1720: SRT1720 5 mg/kg; D-GalN/LPS: D-galactosamine 400 mg/kg + lipopolysaccharide 10 *μ*g/kg; Q + D-GalN/LPS: combination of Q and D-GalN/LPS; SRT1720 + D-GalN/LPS: combination of SRT1720 and D-GalN/LPS. ∗ indicates significant values (*P* ≤ 0.05) compared to the negative control group (vehicle only); # indicates significant values (*P* ≤ 0.05) compared to the D-GalN/LPS group. The results are expressed as means ± SEM, *n* = 5 (courtesy of *Physiological Research*, reference [98]).

Studies have shown that both LPS and ROS can increase the production of heme, which is cytotoxic. It is highly lipophilic and can intercalate into and further peroxidate lipid membranes. As an adaptive mechanism against such toxicity, HO-1 is induced to catabolize heme into iron and less reactive and potentially cytoprotective metabolites, biliverdin and carbon monoxide. Biliverdin is rapidly converted to bilirubin, by bilirubin reductase. Both biliverdin and bilirubin have reducing properties through elusive mechanisms. There is some evidence that these bile pigments directly scavenge free radicals, with bilirubin being a much more potent antioxidant [96]. Furthermore, some authors have proposed an indirect mechanism (biliverdin/bilirubin redox cycle), in which ROS oxidize bilirubin to biliverdin, with ROS themselves being reduced in the process [97]. This explains why the induction of HO-1 by D-GalN/LPS (Figure 2(b)) is accompanied by a proportionate increase in total bilirubin levels [98]. Although not investigated in our studies, HO-1 induction is usually accompanied by concomitant production of carbon monoxide (CO) and iron. Like bilirubin, CO has a cytoprotective role. Among other things, it regulates intracellular calcium ion mobilization, can actively relax hepatic sinusoids [99], and regulates bile canalicular contractility [100]. All these can restore sinusoidal perfusion and bile clearance to promote tolerance against heme toxicity [101]. Unlike bilirubin and CO, free iron is toxic, even at low levels. It catalyzes the formation of ROS via the Fenton and Haber-Weiss reactions [102]. Also, because iron produced from heme degradation can convert hydrogen peroxide into hydroxyl radical, catalase is rapidly induced to quickly terminate the reaction [103]. In all our acute experimental studies, levels of conventional antioxidants catalase (Table 1), HO-1, and bilirubin were markedly increased in response to the hepatotoxin. But their inductions, under the present experimental conditions, were not sufficient to be hepatoprotective. Recently however, it was reported that the endogenous antioxidant catalase delays high-fat diet-induced liver injury in mice [104]. Hence, it is logical to assume that their antioxidant capacities were overwhelmed by the extent of D-GalN/LPS-induced ROS.

### 6.2. D-GalN/LPS Effect on SIRT1

Contrary to other endogenous antioxidants, D-GalN/LPS downregulated SIRT1 expression (Figures 1(c) and 2(a)). Studies have shown that oxidative stress can affect SIRT1 activity at different levels. For instance, ROS can promote interaction of SIRT1 with SENP1 (Sentrin-specific protease 1) desumoylase, leading to p53-induced apoptosis [105]. There is also substantial evidence that ROS can covalently modify SIRT1 and mark it for proteasomal degradation [106]. Furthermore, oxidative stress can induce expression of microRNAs such as miR-34a, which could bind to the 3′UTR of SIRT1 mRNA and directly inhibit SIRT1 translation [107]. Besides gene expression, some studies have shown that oxidative stress can overactivate Poly (ADP-ribose) polymerase (PARP), deplete cellular NAD^+^ stores, and decrease SIRT1 deacetylase activity [108]. From our findings and those of others, ROS deprive organisms of many of the putative SIRT1-mediated health benefits. With this finding, it made sense to explore the therapeutic potential of STACs in oxidative stress pathologies such as hepatotoxicity.

### 6.3. Quercetin and SRT1720 Upregulate SIRT1 and Alleviate D-GalN/LPS-Induced Hepatotoxicity

In this study, we pretreated the D-GalN/LPS-intoxicated animals with a single dose of either 50 mg/kg quercetin or 5 mg/kg SRT1720. Each of these STACs was hepatoprotective and returned the conventional antioxidant enzymes to the baseline (Table 1) but increased SIRT1 expression. There was a remarkably inverse proportion between SIRT1 expression (Figure 2(a)) and liver injury markers (Table 1). As if, the higher the SIRT1 expression, the healthier the liver. How SIRT1 activators increase its expression is still elusive. There are some scanty reports that STACs can positively feedback SIRT1 expression through a FOXO1-mediated mechanism. By activating SIRT1, quercetin and SRT1720 can deacetylate and increase FOXO1's DNA-binding ability. In other words, they can potentiate the transcriptional activity of FOXO1 [109]. In rats, FOXO1 directly activates SIRT1 transcription through binding to the IRS-1 and FKHD-like responsive elements within the SIRT1 promoter region [110]. This autofeedback loop mechanism may partly explain why STACs increase SIRT1 expression in D-GalN/LPS-treated rats.

### 6.4. Inhibiting SIRT1 Blocks the Hepatoprotective Effects of Resveratrol

We also explored the therapeutic potential of resveratrol (2.3 mg/kg) in D-GalN/LPS-induced hepatotoxicity (Figures 1(a)–1(c)). As expected, resveratrol was hepatoprotective. However, contrary to quercetin, resveratrol did not have any significant effect on the SIRT1 protein expression level, alone or in combination with D-GalN/LPS. This could possibly be due to a relatively shorter duration of the experiment (6 hours) or a lower dose of resveratrol. Studies have shown that SIRT1 expression is both time dependent and dose dependent [111]. For example, resveratrol administration (25 mg/kg/day) for 8 weeks significantly improved the expression of SIRT1 mRNA in the hepatic tissue of rats with NAFLD [12]. Similarly, the increased SIRT1 mRNA expression and serum levels were detected in the clinical trial evaluating the 30-day effects of daily resveratrol (500 mg/day) supplementation in healthy slightly overweight individuals [112]. Moreover, SIRT1 protein expression does not always correlate to SIRT1 activity [86]. As originally shown by Howitz, STACs can activate and increase the activity of an individual enzyme, even without having any effect on SIRT1 expression *per se*. To extend the role of SIRT1 catalysis in the hepatoprotective effects of resveratrol, some RES + D − GalN + LPS animals were pretreated with EX-527, which is a highly potent and selective SIRT1 inhibitor. Normally, SIRT1-mediated deacetylation reaction couples lysine deacetylation and NAD hydrolysis to yield nicotinamide and O-acetyl-ADP-ribose. Density analysis suggests that EX-527 blocks catalysis by occupying SIRT1's C-pocket and preventing the release of O-acetyl-ADP-ribose [113]. In our study, EX-527 did not have any significant effect on SIRT1 expression. However, EX-527 obstructed the protective effects of resveratrol and strengthened D-GalN/LPS-induced hepatic injury. This latter finding exclusively confirms that unimpaired SIRT1 catalytic activity is crucial for the liver-protective effects of resveratrol. If SIRT1 is inhibited, then the health benefits of resveratrol are concomitantly blocked.

### 6.5. SIRT1 and Carbon Tetrachloride-Induced Hepatotoxicity after Repeated Doses

Among the well-known hepatotoxins, there is carbon tetrachloride (CTC) that produces liver injury due to a radical production in experimental animals. CTC accrues in hepatocytes, where it is metabolized by the mitochondrial monooxygenase isoform CYP2E1 into a trichloromethyl (CCl_3_∗) reactive substituent [114]. CCl_3_∗ binds to oxygen under creation of the reactionary trichloromethyl peroxyl radical (CCl_3_OO∗) [115]. These ROS can oxidize a wide array of biological molecules, ranging from lipid membranes to DNA. CTC also inhibits calcium ATPases [116] and consequently impairs calcium storage in the subcellular organelles [117]. It is leading to pathologically elevated levels of free cytosolic calcium ions thereby provoking apoptosis and necrosis. Under physiologic conditions, apoptosis is usually evened out by mitosis to preserve standard cell turnover and tissue homeostasis [118]. On the other hand, if it is massive and sustained [119], it may cause liver failure [120]. In our research, the extent of the liver damage, induced by application of 0.5 ml/kg CTC each third day, for two weeks, was confirmed biochemically through the significant increase in various markers of liver injury and oxidative stress and histologically as well [121].

### 6.6. Carbon Tetrachloride Elevates HO-1 and SIRT1 Expressions

**Figure 3:**
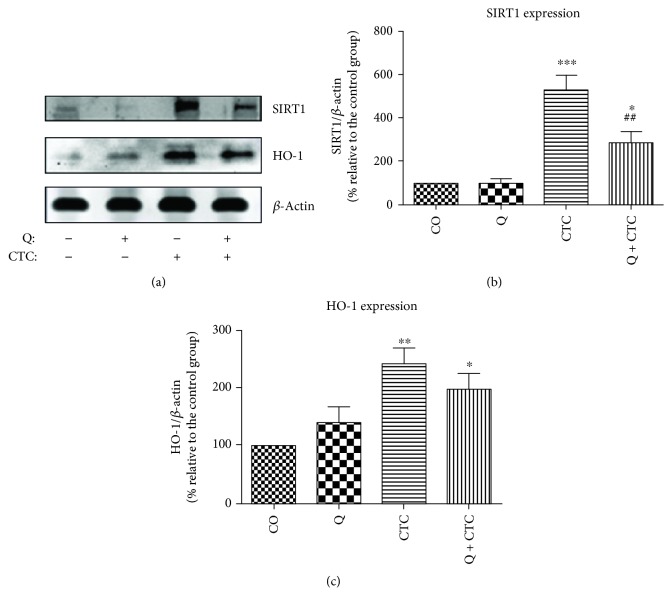
Effects of quercetin and carbon tetrachloride treatments on SIRT1 and heme oxygenase-1 (HO-1) expressions. (a) Representative Western blot image. (b) Quantification of SIRT1 expression by densitometry. (c) Quantification of HO-1 protein expression by densitometry. Beta-actin was used as an endogenous control. CO: negative control, vehicle only; Q: quercetin; CTC: carbon tetrachloride; Q + CTC: quercetin plus carbon tetrachloride. Data are presented as mean ± SEM, *n* = 6. ^∗^*P* < 0.05, ^∗∗^*P* < 0.01, or ^∗∗∗^*P* < 0.001 relative to the CO group (vehicle only). ^##^*P* < 0.01 relative to the CTC group (courtesy of *Elsevier*, reference [121]).

Like D-GalN/LPS, CTC increased HO-1 (Figures 3(a) and 3(c)), bilirubin, and catalase levels (Table 2). Furthermore, CTC increased nitrite levels in plasma (Table 2), indicating enhanced nitric oxide (NO) production [122]. Studies have shown that CTC can stimulate Kupffer cell to release TNF-*α*, which can extend NO production [123]. NO can exacerbate additional CTC-induced liver injury by reacting with superoxide ion to form peroxynitrite anion, which is both oxidizing and nitrating [124]. The NO synthase inhibitor, aminoguanidine, has been demonstrated to decrease the necrogenic effects of CTC in the liver [125]. However, unlike D-GalN/LPS, CTC drastically increased SIRT1 expression (Figures 3(a) and 3(b)). Some studies have shown that persistent oxidative stress could induce FOXO1 expression [126], whose activity is tightly regulated by posttranslational modifications [127]. Phosphorylation, in particular, tends to have opposing effects on the FOXO activity [128]. Historically, it is known that phosphorylation of FOXOs by protein kinase B (AKT/PKB) causes nuclear exclusion and transcriptional inactivation of the FOXO factor [129]. It starts the FOXO to lose grip on the DNA and translocate to the cytoplasm [130]. In the cytoplasm, phosphorylated FOXOs are sequestered by binding to 14-3-3 proteins (these scaffold proteins preclude reentry of FOXO into the nucleus [130]) [131]. Recent evidence suggests that oxidative stress activates a different type of kinase, the c-Jun N-terminal kinases (JNK) (also known as stress-activated protein kinases), which phosphorylate FOXOs on a different site than the AKT, and can, therefore, overrule the negative phosphorylation by AKT [132]. Furthermore, JNK phosphorylates 14-3-3 proteins [133] and disrupts their interactions with the FOXOs [134]. JNK also directly phosphorylates the insulin receptor substrate (IRS) and inhibits the PKB pathway [135]. Although research is still underway to further clarify the relationship between PKB, JNK, and FOXO factors, the current evidence strongly suggests a more general function of JNK towards FOXO activation. Hence, it is possible that persistent oxidative stress induces SIRT1 expression via JNK-dependent FOXO1 activation. SIRT1 can encourage cytoprotection or cell death depending on the magnitude of the insult [26]. Elevated levels of ROS, as reported here on CTC-induced liver injury, can initiate SIRT1-mediated apoptosis. For example, the treatment of some cell lines with resveratrol amplifies the chromatin-associated SIRT1 protein binding on the cIAP-2 promoter region, an effect that correlates with a loss of NF-*κ*B-regulated gene expression and sensitization of cells to TNF-*α*-induced apoptosis [38]. In addition, SIRT1 can direct p53 to the mitochondria where it inactivates antiapoptotic proteins, Bcl-xL and Bcl-2 [40, 136]. This may indicate that the cell-killing effects of CTC are partially SIRT1 mediated.

### 6.7. Quercetin Decreases SIRT1 Expression and Alleviates CTC-Induced Hepatotoxicity

Daily oral treatment with 100 mg/kg quercetin alleviated oxidative stress and mitigated the liver-damaging effects of CTC (Table 2). In spite of not ruling out the direct ROS scavenging properties of quercetin in this study, the involvement of enzyme systems in the cytoprotective effects of quercetin has been reported in numerous studies. For instance, quercetin may interfere with the prooxidant enzymes including iNOS and reduce the release of RNS [137]. Quercetin has also been shown to upregulate numerous antioxidant enzymes [138]. However, in this study, quercetin returned catalase levels back to normal (Table 2) and had no significant effect on HO-1 expression in CTC-treated rats (Figure 3(c)), suggesting that the abovementioned hepatoprotection occurs through alternative mechanisms. Contrary to the previous findings in acute experiments, quercetin reduced SIRT1 expression though to still greater than normal levels (Figure 3(b)). According to the available literature, this is the first report showing that quercetin fine-tunes SIRT1 expression to a lower but still effective level to deal with xenobiotic-induced hepatotoxicity. The mechanism of the downregulatory effect of quercetin on SIRT1 expression remains to be postulated. Considering that SIRT1 overexpression also promotes hepatic tumorigenesis [139], it is plausible to assume that the beneficial health effects of SIRT1 activation occur within a definite range of expression. Hence, a kind of dose expression (response) is needed to fortify this concept.

## 7. Concluding Remarks

The present report supports the fact that oxidative stress is a key factor in hepatotoxicity. As such, the hepatoprotective potency of most drugs is likely to be dictated by their antioxidant capabilities. Conventional endogenous antioxidants such as catalase, HO-1, and bilirubin seem to be consistently induced in hepatotoxicity, but with the extensive liver damage as with the hepatotoxic models in our research, endogenous antioxidants provide little or even no protection. Hence, it may be assumed that their antioxidant capacities were overwhelmed by the extent of the liver damage.

**Figure 4:**
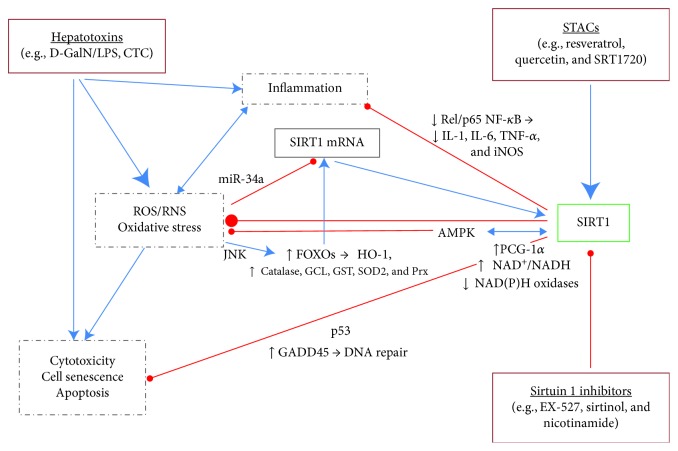
Effects of sirtuin 1-activating compounds (STACs) and inhibitors on chemically induced hepatotoxicity. Hepatotoxins, like D-GalN/LPS and CTC, induce the formation of ROS/RNS in hepatocytes leading to oxidative stress, inflammation, and cell death. SIRT1 is activated and inhibited by stress-responsive factors and plays a dynamic role in regulating cytoprotection and apoptosis depending on the dosing schedule. SIRT1 activation by STACs results to a decrease of cell death and an increase in stress adaptation of hepatocytes due to the activation (↑) or the inhibition (↓) of various signaling and antioxidant molecules (not all included). These hepatoprotective effects can be blocked by administration of SIRT1 inhibitors. Blue →: activation; red ─●: inhibition; AMPK: AMP-activated protein kinase; CTC: carbon tetrachloride; D-GalN: D-galactosamine; FOXOs: forkhead homeobox type O family including FoxO1, FoxO3, and FoxO4; GADD45: growth arrest and DNA damage-inducible protein; GCL: glutamate cysteine ligase; GST: glutathione transferase; HO-1: heme oxygenase-1; IL-1/6: interleukin-1/6; iNOS: inducible nitric oxide synthase; JNK: c-Jun N-terminal kinase; LPS: lipopolysaccharide; miR-34a: microRNA-34a; NAD^+^: nicotinamide adenine dinucleotide; p53: tumor suppressor protein p53; PGC-1*α*: peroxisome proliferator-activated receptor gamma coactivator 1 alpha; Prx: peroxiredoxins; RelA/p65/NF-*κ*B: RelA/p65 subunit of nuclear factor kappa-B; ROS: reactive oxygen species; RNS: reactive nitrogen species; SIRT1: silent information regulator (two) 1 (sirtuin 1); SOD2: superoxide dismutase 2; TNF-*α*: tumor necrosis factor-alpha.

Despite being upregulated by the hepatotoxin, the administered polyphenol either had no significant effect on HO-1 or returned its expression to baseline in the liver of intoxicated rats. Interestingly, the putative stress-responsive enzyme, SIRT1, had differential effects in the liver, depending on the dosing schedule. Like endogenous antioxidants, SIRT1 is profoundly upregulated by the hepatotoxin in repeated dose studies. However, with a single dose of hepatotoxin, the enzyme is downregulated. The optimal SIRT1 expression levels, where there is minimal or no liver damage, are found in rats treated with polyphenols, either alone or in combination with a hepatotoxin. Most importantly, polyphenols tend to fine-tune SIRT1 expression towards normal in the liver of intoxicated rats in both acute and subchronic studies. Together, all these events give an impression that the cytoprotective effects of SIRT1 occur within a limited range of its expression. The catalytic activity of SIRT1 is equally important in the hepatoprotective effects of polyphenols. Synthetic SIRT1 inhibitors block and the allosteric SIRT1 activators mimic the hepatoprotective effects of polyphenols. In conclusion, it is plausible to assume that the beneficial health effects of SIRT1 activation occur within a definite range of expression. Our results and those of others strongly indicate that pharmacologic modulation of SIRT1 by STACs could be a future major step in the treatment of xenobiotic-induced hepatotoxicity (Figure 4).

## Figures and Tables

**Table 1:** Effects of quercetin and SRT1720 pretreatments in lipopolysaccharide-induced hepatitis in D-galactosamine-sensitized (D-GalN/LPS) rats on the levels of AST, ALT, and catalase^¥^ in plasma and conjugated dienes in liver homogenate, after 24 hours.

	ALT (IU/l)	AST (IU/l)	AST : ALT ratio	Catalase^¥^ (nmol/l)	Conjugated dienes (nmol/mg protein)
CO	32 ± 4.03	86.0 ± 18.5	2.17 ± 0.45	41.2 ± 11.6	1.52 ± 0.37
Q	55.4 ± 35.78	118.8 ± 35.9	2.46 ± 1.03	37.8 ± 4.9	1.18 ± 0.32
SRT1720	55 ± 45.83	99.3 ± 79.2	2.21 ± 0.27	29.25 ± 7.2	2.07 ± 1.01
D-GalN/LPS	1307.2 ± 513.38^∗^	329.6 ± 95.5^∗^	0.29 ± 0.11^∗^	130.2 ± 3.5^∗^	3.56 ± 0.89^∗^
Q + D-GalN/LPS	209.6 ± 79.58^∗#^	208.0 ± 45.1^∗#^	1.92 ± 0.45^#^	79.2 ± 16.8^#^	1.53 ± 0.45^#^
SRT1720 + D-GalN/LPS	402 ± 191.92^∗#^	258.0 ± 83.4^∗^	1.03 ± 0.49^#^	99.8 ± 14.2^∗#^	1.88 ± 0.35^#^

CO: negative control, vehicle only; Q: quercetin 50 mg/kg; SRT1720: SRT1720 5 mg/kg; D-GalN/LPS: D-galactosamine 400 mg/kg + lipopolysaccharide 10 *μ*g/kg; Q + D-GalN/LPS: combination of Q and D-GalN/LPS; SRT1720 + D-GalN/LPS: combination of SRT1720 and D-GalN/LPS. ∗ indicates significant values (*P* ≤ 0.05) compared to the negative control group (vehicle only); # indicates significant values (*P* ≤ 0.05) compared to the D-GalN/LPS group. The results are expressed as means ± SEM, *n* = 5-8. ^¥^Included as an endogenous antioxidant (courtesy of *Physiological Research*, reference [98]).

**Table 2:** Effects of quercetin and carbon tetrachloride treatments on levels of TBARS and conjugated dienes in liver homogenate; ALT, AST, indirect bilirubin, nitrites, and catalase^¥^ in plasma after 14 days of repeated treatment.

	CO	Q	CTC	Q + CTC
ALT (IU/l)	42.22 ± 5.35	60.00 ± 12.29	1455.60 ± 398.57^∗∗∗^	533.33 ± 86.05^#^
AST (IU/l)	3.92 ± 0.17	4.57 ± 0.27	76.53 ± 19.46^∗∗∗^	31.92 ± 2.73^#^
Indirect bilirubin (*μ*mol/l)	5.35 ± 0.40	7.10 ± 0.79	10.95 ± 1.45^∗^	10.51 ± 1.66^∗^
TBARS (nmol/mg protein)	315.25 ± 20.83	347.96 ± 41.99	723.22 ± 56.93^∗∗∗^	482.88 ± 18.45^∗##^
Conjugated dienes (nmol/mg protein)	1.77 ± 0.36	1.85 ± 0.40	4.47 ± 1.04^∗^	3.93 ± 0.68
Nitrites (*μ*mol/l)	25.78 ± 1.39	25.18 ± 0.87	40.46 ± 6.20^∗^	26.40 ± 1.48^#^
Catalase^¥^ (nM)	35.22 ± 4.25	32.63 ± 2.16	93.78 ± 9.89^∗∗∗^	39.14 ± 4.70^###^

CO: negative control, vehicle only; Q: quercetin; CTC: carbon tetrachloride; Q + CTC: quercetin plus carbon tetrachloride. Data are presented as mean ± SEM, *n* = 6. ^∗^*P* < 0.05, ^∗∗^*P* < 0.01, or ^∗∗∗^*P* < 0.001 relative to the CO group (vehicle only). ^#^*P* < 0.05, ^##^*P* < 0.01, or ^###^*P* < 0.001 relative to the CTC group. ^¥^Included as endogenous antioxidant (courtesy of Elsevier, reference [121]).

## References

[1] Blachier M., Leleu H., Peck-Radosavljevic M., Valla D. C., Roudot-Thoraval F. (2013). The burden of liver disease in Europe: a review of available epidemiological data. *Journal of Hepatology*.

[2] Rayfield B. Why we need extra liver protection, Sinclair Method. http://www.sinclairmethod.com/index_files/Page563.htm.

[3] Williams R. (2006). Global challenges in liver disease. *Hepatology*.

[4] Sanchez-Valle V., Chavez-Tapia N. C., Uribe M., Mendez-Sanchez N. (2012). Role of oxidative stress and molecular changes in liver fibrosis: A review. *Current Medicinal Chemistry*.

[5] Alonso F. T., Garmendia M. L., de Aguirre M., Searle J. (2010). Mortality trend from liver cirrhosis in Chile from 1990 to 2007. *Revista Médica de Chile*.

[6] Nobili V., Carter-Kent C., Feldstein A. E. (2011). The role of lifestyle changes in the management of chronic liver disease. *BMC Medicine*.

[7] Torres D. M., Williams C. D., Harrison S. A. (2012). Features, diagnosis, and treatment of nonalcoholic fatty liver disease. *Clinical Gastroenterology and Hepatology*.

[8] Farghali H., Kameniková L., Hynie S., Kmonicková E. (2000). Silymarin effects on intracellular calcuim and cytotoxicity: a study in perfused rat hepatocytes after oxidative stress injury. *Pharmacological Research*.

[9] Farghali H., Cerný D., Kameníková L. (2009). Resveratrol attenuates lipopolysaccharide-induced hepatitis in D-galactosamine sensitized rats: Role of nitric oxide synthase 2 and heme oxygenase-1. *Nitric Oxide*.

[10] Glauert H. P., Calfee-Mason K., Stemm D. N., Tharappel J. C., Spear B. T. (2010). Dietary antioxidants in the prevention of hepatocarcinogenesis: A review. *Molecular Nutrition & Food Research*.

[11] Haddad Y., Vallerand D., Brault A., Haddad P. S. (2011). Antioxidant and hepatoprotective effects of silibinin in a rat model of nonalcoholic steatohepatitis. *Evidence-Based Complementary and Alternative Medicine*.

[12] Hajighasem A., Farzanegi P., Mazaheri Z., Naghizadeh M., Salehi G. (2018). Effects of resveratrol, exercises and their combination on *Farnesoid X receptor, Liver X receptor and Sirtuin 1* gene expression and apoptosis in the liver of elderly rats with nonalcoholic fatty liver. *PeerJ*.

[13] Pradhan S. C., Girish C. (2006). Hepatoprotective herbal drug, silymarin from experimental pharmacology to clinical medicine. *Indian Journal of Medical Research*.

[14] Chirumbolo S. (2010). The role of quercetin, flavonols, and flavones in modulating inflammatory cell function. *Inflammation & Allergy - Drug Targets*.

[15] Zhao L., Wu J., Yang J., Wei J., Gao W., Guo C. (2011). Dietary quercetin supplementation increases serum antioxidant capacity and alters hepatic gene expression profile in rats. *Experimental Biology and Medicine*.

[16] Zhou H., Beevers C. S., Huang S. (2011). The targets of curcumin. *Current Drug Targets*.

[17] Cerný D., Lekić N., Váňová K. (2011). Hepatoprotective effect of curcumin in lipopolysaccharide/-galactosamine model of liver injury in rats: Relationship to HO-1/CO antioxidant system. *Fitoterapia*.

[18] Lekić N., Canová N. K., Hořínek A., Farghali H. (2013). The involvement of heme oxygenase 1 but not nitric oxide synthase 2 in a hepatoprotective action of quercetin in lipopolysaccharide-induced hepatotoxicity of D-galactosamine sensitized rats. *Fitoterapia*.

[19] Farghali H., Canová N. K., Zakhari S. (2014). Hepatoprotective properties of extensively studied medicinal plant active constituents: possible common mechanisms. *Pharmaceutical Biology*.

[20] Mazucanti C., Cabral-Costa J., Vasconcelos A., Andreotti D., Scavone C., Kawamoto E. (2015). Longevity Pathways (mTOR, SIRT, insulin/IGF-1) as key modulatory targets on aging and neurodegeneration. *Current Topics in Medicinal Chemistry*.

[21] Nassir F., Ibdah J. A. (2016). Sirtuins and nonalcoholic fatty liver disease. *World Journal of Gastroenterology*.

[22] Shuang R., Rui X., Wenfang L. (2016). Phytosterols and dementia. *Plant Foods for Human Nutrition*.

[23] Dai H., Sinclair D. A., Ellis J. L., Steegborn C. (2018). Sirtuin activators and inhibitors: promises, achievements, and challenges. *Pharmacology & Therapeutics*.

[24] Farghali H., Kemelo M. K., Kameníková L., Kutinová Canová N., Maiese K. (2019). SIRT1 mediates hepatoprotective effects of resveratrol-like compounds in experimental liver injury. *Sirtuin Biology in Medicine: Targeting New Avenues of Care in Development, Aging, and Disease*.

[25] Singh C. K., Chhabra G., Ndiaye M. A., Garcia-Peterson L. M., Mack N. J., Ahmad N. (2018). The role of sirtuins in antioxidant and redox signaling. *Antioxidants & Redox Signaling*.

[26] Raynes R., Brunquell J., Westerheide S. D. (2013). Stress inducibility of SIRT1 and its role in cytoprotection and cancer. *Genes & Cancer*.

[27] Toiber D., Sebastian C., Mostoslavsky R. (2011). Characterization of nuclear sirtuins: molecular mechanisms and physiological relevance. *Handbook of Experimental Pharmacology*.

[28] Bonkowski M. S., Sinclair D. A. (2016). Slowing ageing by design: the rise of NAD^+^ and sirtuin-activating compounds. *Nature Reviews Molecular Cell Biology*.

[29] Kulkarni S. R., Soroka C. J., Hagey L. R., Boyer J. L. (2016). Sirtuin 1 activation alleviates cholestatic liver injury in a cholic acid–fed mouse model of cholestasis. *Hepatology*.

[30] Ding R. B., Bao J., Deng C. X. (2017). Emerging roles of SIRT1 in fatty liver diseases. *International Journal of Biological Sciences*.

[31] Nakamura K., Zhang M., Kageyama S. (2017). Macrophage heme oxygenase-1-SIRT1-p53 axis regulates sterile inflammation in liver ischemia reperfusion injury. *Journal of Hepatology*.

[32] Morris B. J. (2013). Seven sirtuins for seven deadly diseases ofaging. *Free Radical Biology & Medicine*.

[33] Scalbert A., Morand C., Manach C., Rémésy C. (2002). Absorption and metabolism of polyphenols in the gut and impact on health. *Biomedicine & Pharmacotherapy*.

[34] Hori Y. S., Kuno A., Hosoda R., Horio Y. (2013). Regulation of FOXOs and p53 by SIRT1 modulators under oxidative stress. *PLoS One*.

[35] Khader A., Yang W.-L., Kuncewitch M. (2014). Sirtuin 1 activation stimulates mitochondrial biogenesis and attenuates renal injury after ischemia-reperfusion. *Transplantation*.

[36] Li Y., Wu S. (2018). Epigallocatechin gallate suppresses hepatic cholesterol synthesis by targeting SREBP-2 through SIRT1/FOXO1 signaling pathway. *Molecular and Cellular Biochemistry*.

[37] Kobayashi Y., Furukawa-Hibi Y., Chen C. (2005). SIRT1 is critical regulator of FOXO-mediated transcription in response to oxidative stress. *International Journal of Molecular Medicine*.

[38] Yeung F., Hoberg J. E., Ramsey C. S. (2004). Modulation of NF-*κ*B-dependent transcription and cell survival by the SIRT1 deacetylase. *The EMBO Journal*.

[39] Lee Y. S., Kang Y. S., Lee J.-S., Nicolova S., Kim J.-A. (2004). Involvement of NADPH oxidase-mediated generation of reactive oxygen species in the apototic cell death by capsaicin in HepG2 human hepatoma cells. *Free Radical Research*.

[40] Han M.-K., Song E.-K., Guo Y., Ou X., Mantel C., Broxmeyer H. E. (2008). SIRT1 regulates apoptosis and *Nanog* expression in mouse embryonic stem cells by controlling p53 subcellular localization. *Cell Stem Cell*.

[41] Wang Y.-Q., Cao Q., Wang F. (2015). SIRT1 protects against oxidative stress-induced endothelial progenitor cells apoptosis by inhibiting FOXO3a via FOXO3a ubiquitination and degradation. *Journal of Cellular Physiology*.

[42] Hubbard B. P., Sinclair D. A. (2014). Small molecule SIRT1 activators for the treatment of aging and age-related diseases. *Trends in Pharmacological Sciences*.

[43] Bravo L. (1998). Polyphenols: chemistry, dietary sources, metabolism, and nutritional significance. *Nutrition Reviews*.

[44] Pandey K. B., Rizvi S. I. (2009). Plant polyphenols as dietary antioxidants in human health and disease. *Oxidative Medicine and Cellular Longevity*.

[45] Egert S., Wolffram S., Bosy-Westphal A. (2008). Daily quercetin supplementation dose-dependently increases plasma quercetin concentrations in healthy humans. *The Journal of Nutrition*.

[46] Erlund I. (2004). Review of the flavonoids quercetin, hesperetin, and naringenin. Dietary sources, bioactivities, bioavailability, and epidemiology. *Nutrition Research*.

[47] Aherne S. A., O’Brien N. M. (2002). Dietary flavonols: chemistry, food content, and metabolism. *Nutrition*.

[48] Burns J., Yokota T., Ashihara H., Lean M. E. J., Crozier A. (2002). Plant foods and herbal sources of resveratrol. *Journal of Agricultural and Food Chemistry*.

[49] Schultz J. C., Hunter M. D., Appel H. M. (1992). Antimicrobial activity of polyphenols mediates plant-herbivore interactions. *Plant Polyphenols*.

[50] Morazonni P., Bombardelli E. (1995). *Silybum marianum* (*Carduus marianus*). *Fitoterapia*.

[51] Kesavanarayanan K. S., Sathiya S., Ranju V. (2012). In vitro cytotoxic, antioxidative and *α*-glucosidase inhibitory potential of a herbal mixture comprised of *Allium sativum* and *Lagerstroemia speciosa*. *European Review for Medical and Pharmacological Sciences*.

[52] Perez Gutierrez R. M., Anaya Sosa I., Hoyo Vadillo C., Victoria T. C. (2011). Effect of flavonoids from *Prosthechea michuacana* on carbon tetrachloride induced acute hepatotoxicity in mice. *Pharmaceutical Biology*.

[53] Cui C. X., Deng J. N., Yan L. (2017). Silibinin capsules improves high fat diet-induced nonalcoholic fatty liver disease in hamsters through modifying hepatic de novo lipogenesis and fatty acid oxidation. *Journal of Ethnopharmacology*.

[54] Loguercio C., Festi D. (2011). Silybin and the liver: from basic research to clinical practice. *World Journal of Gastroenterology*.

[55] Sampson L., Rimm E., Hollman P. C. H., de Vries J. H. M., Katan M. B. (2002). Flavonol and flavone intakes in US health professionals. *Journal of the American Dietetic Association*.

[56] Bonnefont-Rousselot D. (2016). Resveratrol and cardiovascular diseases. *Nutrients*.

[57] Ferrières J. (2004). The French paradox: lessons for other countries. *Heart*.

[58] Kanner J., Gorelik S., Roman S., Kohen R. (2012). Protection by polyphenols of postprandial human plasma and low-density lipoprotein modification: the stomach as a bioreactor. *Journal of Agricultural and Food Chemistry*.

[59] Rahman T., Hosen I., Islam M. M. T., Shekhar H. U. (2012). Oxidative stress and human health. *Advances in Bioscience and Biotechnology*.

[60] Amararathna M., Johnston M., Rupasinghe H. (2016). Plant polyphenols as chemopreventive agents for lung cancer. *International Journal of Molecular Sciences*.

[61] Almeida S., Alves M. G., Sousa M., Oliveira P. F., Silva B. M. (2016). Are polyphenols strong dietary agents against neurotoxicity and neurodegeneration?. *Neurotoxicity Research*.

[62] Ma Y., Gao W., Wu K., Bao Y. (2015). Flavonoid intake and the risk of age-related cataract in China’s Heilongjiang Province. *Food & Nutrition Research*.

[63] Husain S. R., Cillard J., Cillard P. (1987). Hydroxyl radical scavenging activity of flavonoids. *Phytochemistry*.

[64] Di Meo F., Lemaur V., Cornil J. (2013). Free radical scavenging by natural polyphenols: atom versus electron transfer. *The Journal of Physical Chemistry A*.

[65] Howitz K. T., Bitterman K. J., Cohen H. Y. (2003). Small molecule activators of sirtuins extend *Saccharomyces cerevisiae* lifespan. *Nature*.

[66] Kaeberlein M., McDonagh T., Heltweg B. (2005). Substrate-specific activation of sirtuins by resveratrol. *Journal of Biological Chemistry*.

[67] Beher D., Wu J., Cumine S. (2009). Resveratrol is not a direct activator of SIRT1 enzyme activity. *Chemical Biology & Drug Design*.

[68] Baur J., Mai A., Guarente L. (2012). Revelations into resveratrol’s mechanism. *Nature Medicine*.

[69] Hardie D. G., Ross F. A., Hawley S. A. (2012). AMPK: a nutrient and energy sensor that maintains energy homeostasis. *Nature Reviews Molecular Cell Biology*.

[70] Ruderman N. B., Julia Xu X., Nelson L. (2010). AMPK and SIRT1: a long-standing partnership?. *American Journal of Physiology-Endocrinology and Metabolism*.

[71] Fulco M., Cen Y., Zhao P. (2008). Glucose restriction inhibits skeletal myoblast differentiation by activating SIRT1 through AMPK-mediated regulation of Nampt. *Developmental Cell*.

[72] Zhang J. (2006). Resveratrol inhibits insulin responses in a SirT1-independent pathway. *Biochemical Journal*.

[73] Gescher A. J., Steward W. P. (2003). Relationship between mechanisms, bioavailability, and preclinical chemopreventive efficacy of resveratrol: a conundrum. *Cancer Epidemiology and Prevention Biomarkers*.

[74] Farghali H., Kameníková L. (2017). Targeted drug delivery system: potential application to resveratrol. *Ceska a Slovenska Farmacie*.

[75] Milne J. C., Lambert P. D., Schenk S. (2007). Small molecule activators of SIRT1 as therapeutics for the treatment of type 2 diabetes. *Nature*.

[76] Hubbard B. P., Gomes A. P., Dai H. (2013). Evidence for a common mechanism of SIRT1 regulation by allosteric activators. *Science*.

[77] Hu J., Jing H., Lin H. (2014). Sirtuin inhibitors as anticancer agents. *Future Medicinal Chemistry*.

[78] Villalba J. M., Alcaín F. J. (2012). Sirtuin activators and inhibitors. *BioFactors*.

[79] Andrade R. J., Robles M., Fernández-Castañer A., López-Ortega S., López-Vega M. C., Lucena M. I. (2007). Assessment of drug-induced hepatotoxicity in clinical practice: a challenge for gastroenterologists. *World Journal of Gastroenterology*.

[80] Lee W. (2008). Etiologies of Acute Liver Failure. *Seminars in Liver Disease*.

[81] Bateman D. N., Carroll R., Pettie J. (2014). Effect of the UK’s revised paracetamol poisoning management guidelines on admissions, adverse reactions and costs of treatment. *British Journal of Clinical Pharmacology*.

[82] Bernal W., Auzinger G., Dhawan A., Wendon J. (2010). Acute liver failure. *The Lancet*.

[83] Eren S. H., Demirel Y., Ugurlu S., Korkmaz I., Aktas C., Güven F. M. K. (2010). Mushroom poisoning: retrospective analysis of 294 cases. *Clinics*.

[84] Fleming L. (1995). *Environmental Medicine: Integrating a Missing Element into Medical Education*.

[85] Strader D. B., Bacon B. R., Lindsay K. L. (2002). Use of complementary and alternative medicine in patients with liver disease. *The American Journal of Gastroenterology*.

[86] Wojnarová L., Kutinová Canová N., Farghali H., Kučera T. (2015). Sirtuin 1 modulation in rat model of acetaminophen-induced hepatotoxicity. *Physiological Research*.

[87] Farghali H., Kemelo M. K., Wojnarová L., Canová N. K. (2016). In vitro and in vivo experimental hepatotoxic models in liver research: applications to the assessment of potential hepatoprotective drugs. *Physiological Research*.

[88] Farghali H., Kutinová Canová N., Lekić N. (2013). Resveratrol and related compounds as antioxidants with an allosteric mechanism of action in epigenetic drug targets. *Physiological Research*.

[89] Nolan J. P. (1981). Endotoxin, reticuloendothelial function, and liver injury. *Hepatology*.

[90] Hsu H.-Y., Wen M.-H. (2002). Lipopolysaccharide-mediated reactive oxygen species and signal transduction in the regulation of interleukin-1 gene expression. *Journal of Biological Chemistry*.

[91] Silverstein R. (2004). D-Galactosamine lethality model: scope and limitations. *Journal of Endotoxin Research*.

[92] Liu L.-M., Zhang J.-X., Luo J. (2008). A role of cell apoptosis in lipopolysaccharide (LPS)-induced nonlethal liver injury in D-galactosamine (D-GalN)-sensitized rats. *Digestive Diseases and Sciences*.

[93] Kemelo M. K., Horinek A., Canová N. K., Farghali H. (2016). Comparative effects of quercetin and SRT1720 against D-galactosamine/lipopolysaccharide-induced hepatotoxicity in rats: biochemical and molecular biological investigations. *European Review for Medical and Pharmacological Sciences*.

[94] Parmar K., Singh G., Gupta G., Pathak T., Nayak S. (2016). Evaluation of De Ritis ratio in liver-associated diseases. *International Journal of Medical Science and Public Health*.

[95] Botros M., Sikaris K. A. (2013). The De Ritis ratio: the test of time. *Clinical Biochemist Reviews*.

[96] Jansen T., Hortmann M., Oelze M. (2010). Conversion of biliverdin to bilirubin by biliverdin reductase contributes to endothelial cell protection by heme oxygenase-1—evidence for direct and indirect antioxidant actions of bilirubin. *Journal of Molecular and Cellular Cardiology*.

[97] Baranano D. E., Rao M., Ferris C. D., Snyder S. H. (2002). Biliverdin reductase: a major physiologic cytoprotectant. *Proceedings of the National Academy of Sciences of the United States of America*.

[98] Kemelo M. K., Kutinová Canová N., Horinek A., Farghali H. (2017). Sirtuin-activating compounds (STACs) alleviate D-galactosamine/lipopolysaccharide-induced hepatotoxicity in rats: involvement of sirtuin 1 and heme oxygenase 1. *Physiological Research*.

[99] Suematsu M., Goda N., Sano T. (1995). Carbon monoxide: an endogenous modulator of sinusoidal tone in the perfused rat liver. *Journal of Clinical Investigation*.

[100] Shinoda Y., Suematsu M., Wakabayashi Y. (1998). Carbon monoxide as a regulator of bile canalicular contractility in cultured rat hepatocytes. *Hepatology*.

[101] Kyokane T., Norimizu S., Taniai H. (2001). Carbon monoxide from heme catabolism protects against hepatobiliary dysfunction in endotoxin-treated rat liver. *Gastroenterology*.

[102] Das T. K., Wati M. R., Fatima-Shad K. (2014). Oxidative stress gated by Fenton and Haber Weiss reactions and its association with Alzheimer’s disease. *Archives of Neuroscience*.

[103] Hermes-Lima M. (2005). Oxygen in biology and biochemistry: role of free radicals. *Functional Metabolism: Regulation and Adaptation*.

[104] Piao L., Choi J., Kwon G., Ha H. (2017). Endogenous catalase delays high-fat diet-induced liver injury in mice. *The Korean Journal of Physiology & Pharmacology*.

[105] Yang Y., Fu W., Chen J. (2007). SIRT1 sumoylation regulates its deacetylase activity and cellular response to genotoxic stress. *Nature Cell Biology*.

[106] Caito S., Rajendrasozhan S., Cook S. (2010). SIRT1 is a redox-sensitive deacetylase that is post-translationally modified by oxidants and carbonyl stress. *The FASEB Journal*.

[107] Yamakuchi M., Ferlito M., Lowenstein C. J. (2008). miR-34a repression of SIRT1 regulates apoptosis. *Proceedings of the National Academy of Sciences of the United States of America*.

[108] Braidy N., Guillemin G. J., Mansour H., Chan-Ling T., Poljak A., Grant R. (2011). Age related changes in NAD+ metabolism oxidative stress and Sirt1 activity in Wistar rats. *PLoS One*.

[109] Banks A. S., Kon N., Knight C. (2008). SirT1 gain of function increases energy efficiency and prevents diabetes in mice. *Cell Metabolism*.

[110] Xiong S., Salazar G., Patrushev N., Alexander R. W. (2011). FoxO1 mediates an autofeedback loop regulating SIRT1 expression. *Journal of Biological Chemistry*.

[111] Morita Y., Wada-Hiraike O., Yano T. (2012). Resveratrol promotes expression of SIRT1 and StAR in rat ovarian granulosa cells: an implicative role of SIRT1 in the ovary. *Reproductive Biology and Endocrinology*.

[112] Roggerio A., Strunz C., Pacanaro A. (2018). Gene expression of sirtuin-1 and endogenous secretory receptor for advanced glycation end products in healthy and slightly overweight subjects after caloric restriction and resveratrol administration. *Nutrients*.

[113] Gertz M., Fischer F., Nguyen G. T. T. (2013). Ex-527 inhibits Sirtuins by exploiting their unique NAD^+^-dependent deacetylation mechanism. *Proceedings of the National Academy of Sciences of the United States of America*.

[114] Stoyanovsky D. A., Cederbaum A. I. (1999). Metabolism of carbon tetrachloride to trichloromethyl radical: an ESR and HPLC-EC study. *Chemical Research in Toxicology*.

[115] Mico B. A., Pohl L. R. (1983). Reductive oxygenation of carbon tetrachloride: trichloromethylperoxyl radical as a possible intermediate in the conversion of carbon tetrachloride to electrophilic chlorine. *Archives of Biochemistry and Biophysics*.

[116] Izutsu K. T., Smuckler E. A. (1978). Effects of carbon tetrachloride on rat liver plasmalemmal calcium adenosine triphosphatase. *The American Journal of Pathology*.

[117] Verkhratsky A. (2007). Calcium and cell death. *Sub-Cellular Biochemistry*.

[118] Patel T., Roberts L., Jones B., Gores G. (1998). Dysregulation of apoptosis as a mechanism of liver disease: an overview. *Seminars in Liver Disease*.

[119] Canbay A., Feldstein A. E., Higuchi H. (2003). Kupffer cell engulfment of apoptotic bodies stimulates death ligand and cytokine expression. *Hepatology*.

[120] Guicciardi M. E., Gores G. J. (2005). Apoptosis: a mechanism of acute and chronic liver injury. *Gut*.

[121] Kemelo M. K., Pierzynová A., Kutinová Canová N., Kučera T., Farghali H. (2017). The involvement of sirtuin 1 and heme oxygenase 1 in the hepatoprotective effects of quercetin against carbon tetrachloride-induced sub-chronic liver toxicity in rats. *Chemico-Biological Interactions*.

[122] Song S. Z., Choi Y. H., Jin G. Y., Li G. Z., Yan G. H. (2011). Protective effect of cornuside against carbon tetrachloride-induced acute hepatic injury. *Bioscience, Biotechnology, and Biochemistry*.

[123] Lee C.-H., Park S. W., Kim Y. S. (2007). Protective mechanism of glycyrrhizin on acute liver injury induced by carbon tetrachloride in mice. *Biological & Pharmaceutical Bulletin*.

[124] Rubbo H., Trostchansky A., O’Donnell V. B. (2009). Peroxynitrite-mediated lipid oxidation and nitration: mechanisms and consequences. *Archives of Biochemistry and Biophysics*.

[125] Al-Shabanah O. A., Alam K., Nagi M. N., Al-Rikabi A. C., Al-Bekairi A. M. (1999). Protective effect of aminoguanidine, a nitric oxide synthase inhibitor, against carbon tetrachloride-induced hepatotoxicity in mice. *Life Sciences*.

[126] Gómez-Crisóstomo N. P., Rodríguez Martínez E., Rivas-Arancibia S. (2014). Oxidative stress activates the transcription factors FoxO 1a and FoxO 3a in the hippocampus of rats exposed to low doses of ozone. *Oxidative Medicine and Cellular Longevity*.

[127] Wang Y., Zhou Y., Graves D. T. (2014). FOXO transcription factors: their clinical significance and regulation. *BioMed Research International*.

[128] van den Berg M. C. W., Burgering B. M. T. (2011). Integrating opposing signals toward forkhead box O. *Antioxidants & Redox Signaling*.

[129] Brunet A., Bonni A., Zigmond M. J. (1999). Akt promotes cell survival by phosphorylating and inhibiting a forkhead transcription factor. *Cell*.

[130] Calnan D. R., Brunet A. (2008). The FoxO code. *Oncogene*.

[131] Obsilová V., Silhan J., Boura E., Teisinger J., Obsil T. (2008). 14-3-3 proteins: a family of versatile molecular regulators. *Physiological Research*.

[132] van den Berg M. C. W. (2013). *Complex Regulation of Forkhead Box O Transcription Factors*.

[133] Yoshida K., Yamaguchi T., Natsume T., Kufe D., Miki Y. (2005). JNK phosphorylation of 14-3-3 proteins regulates nuclear targeting of c-Abl in the apoptotic response to DNA damage. *Nature Cell Biology*.

[134] Sunayama J., Tsuruta F., Masuyama N., Gotoh Y. (2005). JNK antagonizes Akt-mediated survival signals by phosphorylating 14-3-3. *The Journal of Cell Biology*.

[135] Lee Y. H., Giraud J., Davis R. J., White M. F. (2003). c-Jun N-terminal Kinase (JNK) mediates feedback inhibition of the insulin signaling cascade. *Journal of Biological Chemistry*.

[136] Moll U. M., Wolff S., Speidel D., Deppert W. (2005). Transcription-independent pro-apoptotic functions of p53. *Current Opinion in Cell Biology*.

[137] Kao T.-K., Ou Y.-C., Raung S.-L., Lai C.-Y., Liao S.-L., Chen C.-J. (2010). Inhibition of nitric oxide production by quercetin in endotoxin/cytokine-stimulated microglia. *Life Sciences*.

[138] Kobori M., Takahashi Y., Akimoto Y. (2015). Chronic high intake of quercetin reduces oxidative stress and induces expression of the antioxidant enzymes in the liver and visceral adipose tissues in mice. *Journal of Functional Foods*.

[139] Hao C., Zhu P.-X., Yang X. (2014). Overexpression of SIRT1 promotes metastasis through epithelial-mesenchymal transition in hepatocellular carcinoma. *BMC Cancer*.

[140] Kemelo M. K., Wojnarová L., Kutinová Canová N., Farghali H. (2014). D-galactosamine/lipopolysaccharide-induced hepatotoxicity downregulates sirtuin 1 in rat liver: role of sirtuin 1 modulation in hepatoprotection. *Physiological Research*.

